# Kinetics of maternally-derived serogroup A, C, Y and W-specific meningococcal immunoglobulin G in Malian women and infants

**DOI:** 10.1016/j.vaccine.2019.03.045

**Published:** 2019-04-24

**Authors:** H. Findlow, M.D. Tapia, S.O. Sow, F.C. Haidara, F. Coulibaly, A.M. Keita, F. Diallo, M. Doumbia, A. Traore, N. Schluterman, D.A. Clark, R. Borrow, M.M. Levine

**Affiliations:** a Vaccine Evaluation Unit, Public Health England, Manchester Royal Infirmary, Manchester, UK; b Center for Vaccine Development, University of Maryland School of Medicine, Baltimore, MD, USA; c Centre pour le Développement des Vaccins, Bamako, Mali; d Department of Epidemiology, University of Maryland, Baltimore, MD, USA; e Department of Epidemiology, Emory University Rollins School of Public Health, Atlanta, GA, USA

**Keywords:** Meningococcal, Maternal immunization, Vaccination, CI, confidence intervals, ELISA, enzyme-linked immunosorbent assay, EPI, expanded program of Immunization, GMC, geometric mean concentration, MCV, quadrivalent meningococcal conjugate vaccine, Men, meningococcal serogroup, mHSA, methylated human serum albumin, SBA, serum bactericidal antibody, TIV, trivalent influenza vaccine

## Abstract

A prospective, randomised, controlled observer-blind trial measuring the efficacy and immunogenicity of trivalent influenza vaccine (TIV) and the immunogenicity of quadrivalent meningococcal conjugate vaccine (MCV) in pregnant women and their infants up to 6 months of age was conducted in Mali. Here we reported the immunogenicity of MCV, which was used as a comparator vaccine to TIV, in this population. Third-trimester pregnant Malian women were randomized to receive TIV or MCV. Blood samples were collected from women prior to vaccination, 28 days post-vaccination, at delivery and 3 and 6 months post-delivery and from infants at birth and 3 and 6 months of age. Meningococcal-specific serogroup (Men) A, C, Y and W-specific antibodies were measured by enzyme linked immunosorbent assay in a randomly selected subset of 50 mother-infant pairs where the mother had received MCV. At birth, 94.0% (47/50) of infants had MenA specific IgG levels ≥ 2 µg/mL decreasing to 72.9% and 30.4% at 3 and 6 months of age. For MenC, 81.3% (39/48) of infants had MenC specific IgG levels ≥ 2 µg/mL at birth decreasing to 29.4% and 17.8% at 3 and 6 months of age. For MenY, 89.6% (43/48) of infants had MenY specific IgG levels ≥ 2 µg/mL at birth decreasing to 64.6% and 62.5% at 3 and 6 months of age. For MenW, 89.6% (43/48) of infants had MenW specific IgG levels ≥ 2 μg/ml at birth decreasing to 62.5% and 41.7% at 3 and 6 months of age.

Maternal immunization with MCV conveyed protective levels of IgG at birth through to 3 months of age in the majority of infants.

## Highlights

• Immunisation with MCV during pregnancy resulted in an antibody response.
• Maternal immunization with MCV conveyed protective levels of MenA IgG at birth.
• Infant antibody levels declined over the first 3 months of life.

## 1 Introduction

Maternal antibodies are transferred from mother to child and protect the neonate and infant during a time of immune maturation. The majority of antibodies which are transferred across the placenta are IgG, and these passively acquired antibodies enter the bloodstream of the offspring providing protection in the same way as actively acquired antibodies. Following birth, these IgG antibodies are present in the bloodstream and are effective in providing protection to the neonate, however these antibodies are present in finite amounts and decline over time.

Infants are not usually immunized before the age of 2–3 months (depending upon country-specific immunization schedules) because of their relative immunological immaturity. Immunization of the mother during pregnancy, at an optimal time can provide protection to the infant earlier in life. Maternally derived antibodies wane over time and the kinetics of this decline is correlated to the amount of maternal antibody present in the neonate after birth. Therefore if high levels of maternal antibodies can be achieved in infants, protection will be afforded during the most immature phase of their immune system.

The principle of maternal immunization is supported by data for vaccination against tetanus, influenza and pertussis [1], [2], [3], [4]. Immunization with the acellular pertussis vaccine has proven to increase the level of maternal antibodies and protect infants from clinical pertussis. Maternal pertussis vaccination was introduced in the U.K. in 2012, in response to an increase in infant deaths. This maternal vaccination program impacted on infant pertussis, with vaccine effectiveness being >90% for those infants who’s mother received a pertussis vaccine at least 1 week prior to delivery [3], [4]. In principle maternal immunization can be applied to other vaccines and infectious diseases. However, it has been reported that maternal immunization with a pneumococcal polysaccharide vaccine does not protect infants against clinical disease [5].

A recent post-licensure clinical trial of the safety, immunogenicity and efficacy of maternal influenza immunization for prevention of influenza in infants younger than 6 months has recently been conducted [6]. A quadrivalent meningococcal conjugate vaccine was chosen as a comparator vaccine for this trial and provided the opportunity to investigate mother and infant responses to meningococcal conjugate vaccination during pregnancy. This trial was conducted with Mali which is located within the sub-Saharan meningitis belt.

## 2 Methods

The full study details have been reported previously [6]. In brief, this prospective, active-controlled, observer-blind, randomized phase 4 trial was conducted 2011 to 2014 in Bamako, Mali. Pregnant women who were ≥28 weeks gestation were eligible for enrollment. Those women who met the inclusion criteria [6] and consented for participation were randomly allocated to receive trivalent inactivated influenza vaccine (TIV) (Vaxigrip, Sanofi Pasteur, Lyon, France) or quadrivalent meningococcal conjugate vaccine (MCV) (Menactra, Sanofi Pasteur, Lyon, France).

Quadrivalent meningococcal conjugate vaccine, composed of 4 μg each of Neisseria meningitidis serogroup A, C, Y, and W polysaccharides conjugated to diphtheria toxoid protein, was supplied in single-dose vials. A single 0·5 mL dose of trivalent inactivated influenza vaccine or quadrivalent meningococcal conjugate vaccine was injected into the deltoid muscle. Study vaccines were stored in secure, temperature-monitored refrigerators or cold rooms at 2–8 °C.

From the cohort that received MCV, fifty women were randomly selected for assessment of the immune response to meningococcal vaccination. Blood samples were collected from the mother prior to vaccination and 28 days post vaccination, at delivery and at 3 and 6 months post-partum. Cord blood sample was collected at birth and infant blood samples collected at 3 and 6 months of age.

Maternal immunization history was obtained at the time of study enrollment and 40/50 (80%) reported that they had previously received serogroup A conjugate vaccine (MenAfriVac) during the 2011 vaccination campaign conducted in Mali [12].

This trial is registered with ClinicalTrials.gov, number NCT01430689.

### 2.1 Immunogenicity

Meningococcal (Men) serogroup A, C, W and Y-specific IgG responses were determined using a standardized enzyme-linked immunosorbent assay (ELISA) [7], [8], except that the reference serum CDC1992 and monoclonal-pan antihuman IgG Fc labeled with horseradish peroxidise were used. For the reference serum, CDC1992 was used with the previously assigned serogroup A, C, W and Y-specific IgG concentrations [9], [10]. The polysaccharide and methylated human serum albumin (mHSA) concentrations used for microtitre plate coating were 5 µg/mL of both polysaccharide and mHSA for MenA and MenC specific IgG detection and 2 µg/mL and 1 µg/mL of polysaccharide and mHSA, respectively for MenW and MenY-specific IgG detection. All blood samples were analyzed in the serogroup A ELISA, and samples collected from the mother prior to and 28 days following vaccination and cord blood and infant samples at 3 and 6 months were analyzed in the serogroup C, W and Y ELISA. For each time point, serogroup A, C, W and Y-specific IgG geometric mean concentrations (GMCs) with 95% confidence intervals (CI) were calculated. The percentage of subjects, with 95% confidence intervals (CI) with serogroup A, C, W and Y-specific IgG concentrations ≥2 µg/mL were calculated for each time point. A level of 2 µg/mL was chosen as this is the best threshold of serogroup-specific IgG correlating to protection [11].

### 2.2 Selection of study participants

**Fig. 1:**
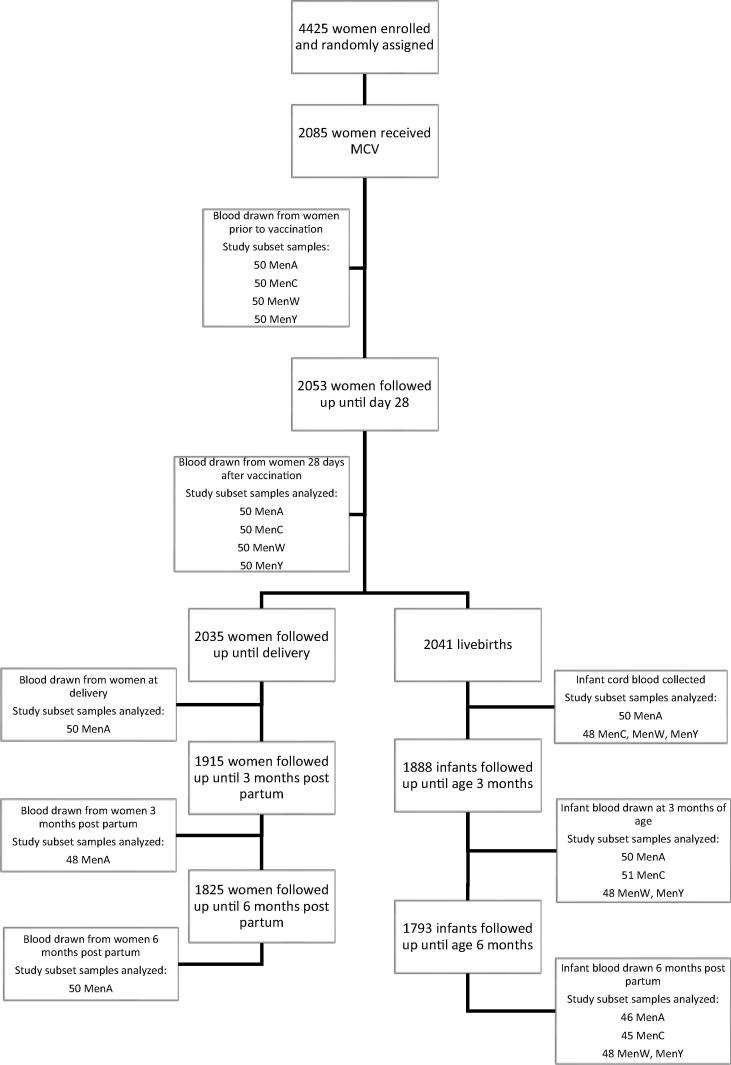
Selection of participants for MCV evaluation.

From the cohort that received MCV, fifty mother-child pairs were selected for assessment of the immune response to meningococcal vaccination, via simple random selection (Fig. 1). Mother-child pairs were eligible for selection if they were missing no more than one mother (out of five) and one child (out of three) blood sample. Blood samples were collected from the mother prior to vaccination and 28 days post vaccination, at delivery and at three and six month’s post-partum. Cord blood sample was collected at birth and infant blood samples collected at three and six months of age.

## 3 Results

Baseline characteristics of mothers randomized to receive MCV were similar to the overall study Cohort (Table 1).

**Table 1:** Baseline characteristics of all mothers randomized to receive MCV.

	All mothers randomized to receive MCV (n = 2085)	Study subset (n = 50)
Age (Years)	24.7 (5.1)	23.7 (5.2)
Gravidity	3.3 (2.1)	3.0 (2.2)
Parity	2.1 (2.0)	1.9 (2.0)
Gestational age at enrolment (weeks)	32.7 (4.6)	32.5 (3.7)

Available method to calculate gestational age at enrolment		
Early ultrasound (<15 weeks)	285 (14%)	9 (18%)
Ultrasound (≥15 weeks)	638 (30%)	13 (26%)
Date last menstrual period	101 (48%)	4 (8)
Uterine height	1061 (51%)	24 (48%)
Completed HIV testing	716 (34%)	17 (34%)
Time from vaccination to delivery (days)	53.5 (28.3)	58.9 (24.8)
Delivered at health centre^a^	1988 (98%)	50 (98%)
Delivery by caesarean section	126 (6%)	4 (8%)
Livebirths^b^	2041 (98%)	51 (100%)
Twin births^a^	36 (2%)	1 (2%)

a Percent based on livebirths.

b Percent based on total births.

### 3.1 Serogroup A

MenA-specific IgG concentrations at each time point measured in samples collected from mother and infants are reported in Table 2 along with the percentage of subjects with MenA-specific IgG ≥ 2 µg/mL.

**Table 2:** Serogroup A-specific IgG GMCs with 95% CI measured in mother samples collected pre- and 28 days post-vaccination, at delivery and 3 and 6 months post partum, cord blood and infant samples collected at 3 and 6 months of age.

Source	Timepoint	n	GMC (−/+ 95%CI)	% of Subjects ≥ 2 µg/mL (−/+ 95%CI)
Mother	Pre-vaccination	50	11.92 (8.44–16.82)	88.0 (75.7–95.5)
	4 weeks post-vaccination	50	40.19 (32.12–50.28)	100.0 (92.9–100.0)
	Delivery	50	29.62 (23.07–38.02)	100.0 (92.9–100.0)
	3 months post-partum	48	27.41 (20.35–36.93)	97.9 (88.9–99.9)
	6 months post-partum	50	31.69 (23.81–42.16)	98.0 (89.4–99.9)

Infant	Cord	50	16.31 (11.79–22.56)	94.0 (83.5–98.7)
	3 months of age	50	3.21 (2.56–4.04)	72.0 (57.5–83.8)
	6 months of age	46	1.41 (1.0–1.98)	30.4 (17.7–45.8)

CI, Confidence interval; GMC, Geometric mean concentration.

Prior to vaccination, the mothers GMC was 11.92 µg/mL (95% CI: 8.44–16.82) which increased to 40.19 µg/mL (95% CI: 32.12–50.28) 28 days post-vaccination with a decline to 29.62 µg/mL (95% CI: 23.07–38.02) at delivery and maintained over the following six months with GMCs of 27.41 µg/mL (95% CI: 20.35–36.93) and 31.69 µg/mL (95% CI: 23.81–42.16) at 3 and 6 months, respectively.

The MenA-specific IgG concentration in the cord blood was approximately half of that measured in the mother at delivery, with GMCs of 16.31 µg/mL (95% CI: 11.79–22.56) and 29.62 µg/mL (95% CI: 23.07–38.02), respectively. Infant MenA-specific IgG antibody levels waned over the first months of life to 3.21 µg/mL (95% CI: 2.56–4.04) at 3 months of age with further waning to 1.41 µg/mL (95% CI: 1.0–1.98) at 6 months of age.

A high proportion of mothers (88.0% (95% CI: 75.7–95.5) had MenA-specific IgG ≥ 2 µg/mL prior to receiving vaccination which increased to 100% (95% CI: 92.9–100.0) 28 days post-vaccination and remained high over the following months (Table 2). A high percentage of cord blood samples had MenA-specific IgG ≥ 2 µg/mL, 94.0% (95% CI: 83.5–98.7) which declined to 72.0% (95% CI: 57.5–83.8) and 30.4% (95% CI: 17.7–45.8) in infants samples taken at 3 and 6 months of age, respectively.

### 3.2 Serogroup C, W and Y

Serogroup C, W and Y-specific IgG GMCs at each time point measured in samples collected from mother and infants are reported in Table 3 along with the percentage of subjects with MenC, W and Y-specific IgG ≥ 2 µg/mL.

**Table 3:** Serogroup C, W and Y-specific IgG GMCs with 95% CI measured in mother samples collected prior to and 28 days post-vaccination, at delivery and 3 and 6 months post partum, cord blood and infant samples collected at 3 and 6 months of age.

Source	Timepoint	MenC			MenW			MenY		
		n	GMC (−/+ 95%CI)	% ≥2 µg/mL (−/+ 95%CI)	n	GMC (−/+ 95%CI)	% ≥2 µg/mL (−/+ 95%CI)	n	GMC (−/+ 95%CI)	% ≥2 µg/mL (−/+ 95%CI)
Mother	Pre-vaccination	50	2.79 (1.87–4.17)	60.0 (45.2–73.6)	50	1.49 (1.09–2.04)	32.0 (19.5–46.7)	50	2.49 (2.12–2.92)	66.0 (51.2–78.8)
	4 weeks post-vaccination	50	12.67 (9.03–17.80)	92.0 (80.8–97.8)	50	48.13 (31.86–72.71)	92.0 (80.8–97.8)	50	33.97 (23.43–49.25)	90.0 (78.2–96.7)

Infant	Cord	48	6.01 (3.99–9.05)	81.3 (67.4–91.1)	48	19.30 (12.11–30.75)	89.6 (77.3–96.5)	48	14.73 (9.89–21.95)	89.6 (77.3–96.5)
	3 months of age	51	1.01 (0.73–1.40)	29.4 (17.5–43.8)	48	4.17 (2.83–6.16)	62.5 (47.4–76.0)	48	4.13 (3.04–5.61)	64.6 (49.5–77.8)
	6 months of age	45	0.47 (0.31–0.73)	17.8 (8.0–32.1)	48	1.67 (1.18–2.38)	41.7 (27.6–56.8)	48	3.07 (2.34–4.02)	62.5 (47.5–76.0)

CI, Confidence interval; GMC, Geometric mean concentration; Men, Meningococcal serogroup.

Prior to vaccination, the MenC, W and Y-specific IgG GMCs were 2.79 µg/mL (95% CI: 1.87–4.17), 1.49 µg/mL (95% CI: 1.09–2.04), and 2.49 µg/mL (95% CI: 2.12–2.92) respectively. One month following vaccination, these levels increased to 12.67 µg/mL (95% CI: 9.03–17.80), 48.13 µg/mL (95% CI: 31.86–72.71), and 33.97 µg/mL (95% CI: 23.43–49.25), respectively. The MenC, W and Y-specific IgG GMCs measured in the cord blood were approximately 40–47% of that present in the mother 28 days post-vaccination with concentrations of 6.01 µg/mL (95% CI: 3.99–9.05), 19.30 µg/mL (95% CI: 12.11–30.75), and 14.73 µg/mL (95% CI: 9.89–21.95), respectively. Antibodies to all three serogroups waned over the first few months of life to GMCs of 1.01 µg/mL (95% CI: 0.73–1.40), 4.17 µg/mL (95% CI: 2.83–6.16), and 4.13 µg/mL (95% CI: 3.04–5.61), for MenC, W and Y, respectively.

Prior to vaccination and 28 days post-vaccination, the proportion of mothers with MenC-specific IgG concentrations ≥ 2 µg/mL were 60.0% (95% CI: 45.2–73.6) and 92.0% (95% CI: 80.8–97.8), respectively. The proportion of cord blood samples with MenC-specific IgG concentrations ≥ 2 µg/mL were 81.3% (95% CI: 67.4–91.1) and the proportion of infants with MenC-specific IgG concentrations ≥ 2 µg/mL at 3 and 6 months of age were 29.4% (95% CI: 17.5–43.8) and 17.8% (95% CI: 8.0–32.1), respectively.

Prior to vaccination and 28 days post-vaccination, the proportion of mothers with MenW-specific IgG concentrations ≥ 2 µg/mL were 32.0% (95% CI: 19.5–46.7) and 92.0% (95% CI: 80.8–97.8), respectively. The proportion of cord blood samples with MenW-specific IgG concentrations ≥ 2 µg/mL were 89.6% (95% CI: 77.3–96.5) and the proportion of infants with MenW-specific IgG concentrations ≥ 2 µg/mL at 3 and 6 months of age were 62.5% (95% CI: 47.4–76.0) and 41.7% (95% CI: 27.6–56.8), respectively.

Prior to vaccination and 28 days post-vaccination, the proportion of mothers with MenY-specific IgG concentrations ≥ 2 µg/mL were 66.0% (95% CI: 51.2–78.8) and 90.0% (95% CI: 78.2–96.7), respectively. The proportion of cord blood samples with MenY-specific IgG concentrations ≥ 2 µg/mL were 89.6% (95% CI: 77.3–96.5) and the proportion of infants with MenY-specific IgG concentrations ≥ 2 µg/mL at 3 and 6 months of age were 64.6% (95% CI: 49.5–77.8) and 62.5% (95% CI: 47.5–76.0), respectively.

## 4 Discussion

Malian women immunised with a serogroup A, C, W and Y conjugate vaccine during the third trimester of pregnancy showed a good antibody response to all four serogroups one month post-vaccination, with all subjects having a serogroup A-specific IgG concentrations ≥ 2 µg/mL and ≥ 90% of subjects ≥ 2 µg/mL for serogroups C, W and Y. Prior to vaccination, a high proportion of mothers had serogroup A-specific IgG concentrations ≥ 2 µg/mL, due to 40 mothers having previously received serogroup A containing vaccines. The serogroup A conjugate vaccine (MenAfriVac) campaign was performed in Mali in 2011 where vaccine was offered to all those aged 1–29 years of age [12]. Serogroup A-specific IgG GMC measured in the mother at delivery was high, with all mothers having a serogroup A-specific IgG ≥ 2 µg/mL. A serogroup A-specific Ig concentration of ≥2 µg/mL is the only parameter validated in efficacy studies [11] and can therefore be considered a putative correlate of protection against serogroup A. This threshold does have limitations as was for meningococcal polysaccharide vaccine and was a measure of total Ig rather than IgG [11].

Maternal antibodies contribute to the protection of neonates and infants from infectious diseases during the first months of life [1], [2], [3], [4]. If meningococcal conjugate vaccines are included in infant immunization schedules they are usually administered at the age of 2–4 months, depending upon the country. In sub-Saharan African countries, MenAfriVac is being introduced into the expanded program immunization (EPI) schedule but only from 9 months of age [13]. MenAfriVac was introduced into the Malian EPI schedule in 2017. This results in infants being dependent for protection against meningococcal disease during their first few months of life, on maternal antibodies which have been transferred across the placenta. The concentration of antibody transferred depends on the serum antibody concentration in the mother, placental function, and the gestational age at birth. The newborn IgG antibody levels usually correlate with maternal levels however the IgG binding to the neonatal Fc receptor (FcRn) can be saturated and therefore the amount transferred depends upon the number of cell surface receptors. In addition, differences may exist between serogroups in the rate of transfer of antibody across the placenta. The MenA-specific IgG measured in cord blood was approximately half that measured in the maternal serum at infant delivery. MenC, W and Y-specific IgG concentrations were not measured in the maternal serum at birth but the cord blood concentrations were approximately 40% of the maternal level one month post-vaccination. In our study, the infant antibody levels declined over the first 3 months of life for all four serogroups with the fold decline varying 3.5–6 fold; with a further reduction over the following three months. The rate of antibody decay observed in this study is similar to those previously reported for maternally acquired antibody [14], [15].

The concentration of antibodies placentally transferred from mother to infant depends on the type of antigen being either protein or polysaccharide, and the subclass of antibodies induced. IgG_1_ antibodies are transferred preferentially across the placenta, with IgG_2_ less efficiently transferred. The response to MCV in adults has been reported to consist of both IgG_1_ and IgG_2_ [16], [17], with the subclass of antibody stimulated following vaccination dependent upon pre-existing IgG subclass present. This population of Malian women is likely to have received prior meningococcal vaccination in response to previous outbreaks and later during the mass campaigns of MenAfriVac vaccination and as a result the response to MCV in this cohort would likely consist of mixed IgG subclasses and hence impacts on the concentration transferred across the placenta.

Protection from meningococcal disease is known to rely upon circulating antibodies. The serum bactericidal antibody (SBA) assay is the correlate of protection for meningococcal disease and if the infants in this study are protected is unknown, thus future studies using the SBA assay would provide this information. This study has shown the potential for maternally immunisation with a meningococcal conjugate vaccine to provide protection to the infant in the first few months of life before they are eligible for vaccination. The administration of conjugate vaccines in pregnancy also offers the potential to boost antibody responses to carrier proteins such as tetanus toxoid which would be advantageous in countries with high rates of neonatal tetanus. Implementation of maternal immunization programmes requires consideration of the potential to reduce infant responses to infant immunizations.

Maternal immunization in the third trimester of pregnancy with MCV has demonstrated the increase of MenA, C, W and Y-specific IgG antibodies in the mother and infant and this approach has the potential to provide protection to the infant early in life.
